# Dual CCR5/CCR2 targeting: opportunities for the cure of complex disorders

**DOI:** 10.1007/s00018-019-03255-6

**Published:** 2019-08-03

**Authors:** Laura Fantuzzi, Maria Tagliamonte, Maria Cristina Gauzzi, Lucia Lopalco

**Affiliations:** 1 National Center for Global Health, Istituto Superiore di Sanità, Rome, Italy; 2 Cancer Immunoregulation Unit, Istituto Nazionale Tumori— IRCCS—“Fond G. Pascale”, Naples, Italy; 3 Immunobiology of HIV Unit, Division Immunology, Transplantation and Infectious Diseases, San Raffaele Scientific Institute, Milan, Italy

**Keywords:** AIDS, Autoimmunity, Liver disease, Neuroinflammation, Therapeutic antibody, Chemokine receptor antagonist

## Abstract

The chemokine system mediates acute inflammation by driving leukocyte migration to damaged or infected tissues. However, elevated expression of chemokines and their receptors can contribute to chronic inflammation and malignancy. Thus, great effort has been taken to target these molecules. The first hint of the druggability of the chemokine system was derived from the role of chemokine receptors in HIV infection. CCR5 and CXCR4 function as essential co-receptors for HIV entry, with the former accounting for most new HIV infections worldwide. Not by chance, an anti-CCR5 compound, maraviroc, was the first FDA-approved chemokine receptor-targeting drug. CCR5, by directing leukocytes to sites of inflammation and regulating their activation, also represents an important player in the inflammatory response. This function is shared with CCR2 and its selective ligand CCL2, which constitute the primary chemokine axis driving the recruitment of monocytes/macrophages to inflammatory sites. Both receptors are indeed involved in the pathogenesis of several immune-mediated diseases, and dual CCR5/CCR2 targeting is emerging as a more efficacious strategy than targeting either receptor alone in the treatment of complex human disorders. In this review, we focus on the distinctive and complementary contributions of CCR5 and CCR2/CCL2 in HIV infection, multiple sclerosis, liver fibrosis and associated hepatocellular carcinoma. The emerging therapeutic approaches based on the inhibition of these chemokine axes are highlighted.

## Introduction

The chemokine system is a key regulator of leukocyte trafficking during immune and inflammatory responses. It also controls survival and effector functions of immune cells as well as processes such as organogenesis, angiogenesis, haematopoiesis, fibrosis and tissue remodeling [1]. Alterations of chemokines and their receptors have been described in pathological processes, including inflammatory and autoimmune diseases, transplant rejection, tumor growth, and metastasis. In addition, some pathogens can interfere with the host chemokines/chemokine receptors network either for host cell entry or for promoting their own survival [2]. Not surprising, these molecules represent the largest target family in modern pharmacology [3].

CCR5 and CCR2 are structurally related chemokine receptors whose genes share significant sequence homology (73%), probably arising from a gene duplication event. CCR5 is expressed on a broad range of cells, including T lymphocytes, macrophages, granulocytes, dendritic cells (DC), microglia, astrocytes, neurons, fibroblasts, and also on epithelium, endothelium, and vascular smooth muscle [4]. Conversely, CCR2 expression is relatively restricted to certain cell types, mainly monocytes, NK and T lymphocytes, though it can be induced in other cells under inflammatory conditions. CCR2 is mainly considered pro-inflammatory, but anti-inflammatory roles have been described in particular cell types such as regulatory T lymphocytes [5].

CCR5 binds with high affinity to CCL3, CCL4, CCL5, CCL3L1, CCL8, CCL11, CCL13, CCL14, CCL16 (agonists), and CCL7 (antagonist) [4]. CCR2 also binds to several chemokines (i.e., CCL2, CCL7, CCL8, CCL12, and CCL13), but CCL2 is the most potent and the only selective ligand. Monocytes/macrophages are the major source of CCL2. Other cells, including DCs, endothelial, epithelial, fibroblast, smooth muscle, astrocytic, microglial and mesangial cells may produce CCL2 either constitutively or in response to several mediators [5–7].

CCR2 can form homo- or heterodimers with other chemokine receptors. Homodimerization may be necessary for CCR2 chemotactic activity and occurs in the absence of the ligand, although it is favored by CCL2 dimers. CCR2/CCR5 heterocomplexes activate calcium response and support cell adhesion rather than chemotaxis, whereas CCR2/CXCR4 heterodimers have an allosteric trans-inhibitory effect on CCL2 binding. Heterodimerization may influence drug selectivity, because the specific antagonist of one receptor may cross-inhibit the other one [8].

CCR5 and CCR2 are key players in the trafficking of lymphocytes and monocytes/macrophages and have been implicated in the pathophysiology of a number of diseases, including viral infections and complex disorders with an inflammatory component. Accordingly, genetic polymorphisms in their coding or regulatory regions, which affect receptor expression or function, have been shown to influence the incidence and/or the course of HIV infection and inflammatory diseases [5, 9–11]. While recent reviews have described the role of CCR5 or CCR2 in human pathological conditions [4, 5, 9, 12], this review is focused on the involvement of both receptors in HIV infection, multiple sclerosis (MS), liver fibrosis and associated hepatocellular carcinoma (HCC), in which these chemokine axes both represent essential component of the pathological processes. We highlight the differences/similarities in their role, discuss their simultaneous blockade as a comprehensive therapeutic perspective for at least some of these multifaceted disorders, and propose new avenues for the future development of more successful therapies.

## CCR5 and CCR2/CCL2 in the pathogenesis of HIV infection

### CCR5 as a gateway for HIV infection of target cells

**Fig. 1:**
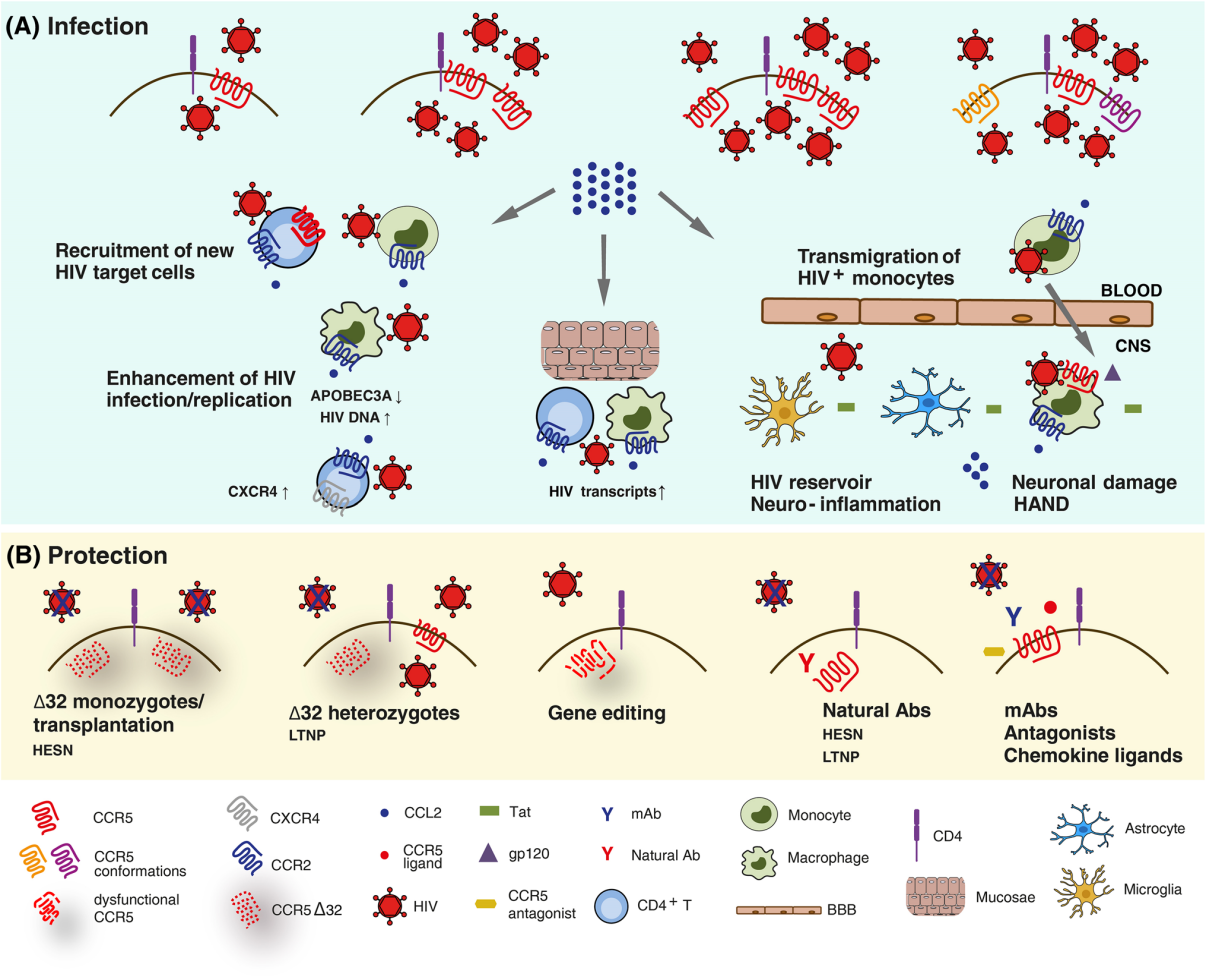
Schematic view of the role of CCR5 and CCR2/CCL2 in the pathogenesis of HIV infection. **a** Infection by HIV occurs when the virus attaches to a susceptible cell and fuses with the cell membrane. The players in this process are the CD4 receptor and a co-receptor, mainly CCR5. The level of viral infection correlates with the number of CCR5 molecules expressed on cell surface, independently from their conformation. HIV-infected cells then release CCL2, which enhances HIV replication with cell type-dependent mechanisms, recruits new HIV target cells and mediates the transmigration of HIV-infected monocytes into the central nervous system (CNS) across the blood–brain barrier (BBB), thus contributing to neuroinflammation, neuronal damage and HIV-associated neurocognitive disorders (HAND). **b** Individuals homozygous for the CCR5Δ32 allele are protected against HIV infection, whereas those heterozygous for CCR5Δ32 progress slowly toward the disease. Natural Abs to CCR5 also  confer protection against HIV infection, as they have been found in HIV-exposed seronegative (HESN) subjects and long-term non-progressors (LTNP). Pharmacological blockade of CCR5 through chemical antagonists, chemokine ligands, mAbs, and gene editing may as well protect from HIV infection

CCR5 is the main co-receptor used by HIV to infect new target cells (Fig. 1a) and it plays a crucial role in HIV mucosal transmission, which accounts for more than 95% of new infections worldwide [13]. The female reproductive tract (FRT), which accounts for around 40% of all HIV transmissions [14], has been largely studied over the past years to better understand sexual HIV transmission and to develop strategies to prevent mucosal HIV acquisition. Compared to heterosexual men, women have bigger mucosal surfaces, a very high level of CCR5 expression on their genital cells and specific pro-inflammatory immune environment; all these factors make FRT more susceptible to HIV infection [15]. The selective mucosal transmission of CCR5-dependent (R5) HIV strains is due to several “gatekeepers” which protect against CXCR4-dependent (X4) (the other main co-receptor) HIV transmission. These include: mucus in the endocervix may trap X4 viruses through various mechanisms (e.g., production of the CXCR4 binding chemokine SDF-1), epithelial cells express CCR5 but not CXCR4, Langerhans cells can be infected by R5 viruses, HIV passes easily through mucosal epithelium if genital ulcerative disease or abrasion is present in genital ulcerative disease or abrasion, macrophages can be preferentially infected by R5 viruses, DCs can be infected by R5 viruses and can trap HIV via DC-SIGN [16].

The evidence of the crucial role of CCR5 in HIV pathogenesis came from the discovery of the Δ32 allele, a 32 base pair deletion in the coding region of the CCR5 gene, which results in functional or non-functional phenotypes depending on its allelic status. Individuals homozygous for this mutation lack functional CCR5 on the cell surface, and are almost completely resistant to HIV infection (Fig. 1b). Homozygosis for CCR5Δ32 was indeed found to be associated with the resistance to infection of HIV-exposed seronegative (HESN) subjects [17]. Conversely, heterozygous individuals, which display decreased cell surface CCR5, are not completely protected, but progress slowly toward the disease [18]. Another CCR5 polymorphism (the so-called -2459 A/G or -59029 A/G), which is located within the gene promoter region, was linked to the level of CCR5 expression and the magnitude of HIV replication in vitro and was associated with a more rapid disease progression in vivo [19, 20]. Notably, CCR5-depleted cell therapy was exploited as functional cure in the “Berlin patient”, who received allogeneic bone marrow transplantation from a CCR5Δ32 homozygous subject and did not have detectable viremia since then [21]. He represented the only subject with a documented HIV cure until the very recent report of the so-called “London patient”, a second case of sustained HIV remission following CCR5Δ32/Δ32 haematopoietic stem-cell transplantation [22]. These observations are good examples of the beneficial effect of CCR5-based approaches for the long-term control of HIV in the absence of antiretroviral therapy.

The number of CCR5 molecules expressed on the membrane of different T-cell subsets is crucial for the susceptibility to HIV infection. CCR5 is expressed at very low level on naïve T cells and at high level on central memory (CM), transitional memory and effector memory (EM) CD4^+^ T lymphocytes [23]. However, a subset of EM cells is relatively resistant to HIV due to post-entry block mechanisms [24]. In HIV-infected CD8^+^ T cells, CCR5 expression is increased by immune activation. Accumulation and increased proliferation of CCR5^+^CD8^+^ EM cells in the inflamed tissues are due to chemokines, such as RANTES, thus CCR5 may drive the migration of such T-cell subset to inflammatory and secondary lymphoid tissues where HIV replicates [25]. CCR5 expression is also dependent of T-cell differentiation and activation. Its levels progressively decrease from T helper (Th) 1, Th17, Th2 and T-follicular helper (Tfh) cells, with the two latter expressing very low levels. Despite Tfh cells almost being CCR5^−^, they represent a relevant T-cell compartment of both latent and replicative virus [26], possibly due to the contribution of CCR5^+^ Tfh precursors to the pool of infected cells. Finally, environmental conditions may influence CCR5 expression as well. Indeed, it was shown that in Africa, parasitic infections elicit immune activation with increased CCR5 expression, which could be responsible of the high HIV infection rate [27].

HIV activates CCR5 in a similar way to chemokines, although it targets a different set of CCR5 conformations. In particular, chemokines bind CCR5 with high or low affinity when it is, respectively, associated to G proteins or not activated. Conversely, HIV recognizes CCR5 with similar affinity either when CCR5 is or is not activated [28]. Moreover, CCR5 regulation is cell type specific and this could explain why chemokines are weak HIV entry inhibitors in macrophages compared with T lymphocytes. This finding may account for CCR5 conformational heterogeneity and explain ligand- and cell type-specific sensitivity of receptor down-regulation [29].

Interestingly, natural antibodies (Abs) to CCR5 were found in several cohorts of HESN subjects [30]. These Abs recognize the first extracellular loop of CCR5 and do not interfere with HIV binding, which takes place in the N-terminus and the second extracellular loop. However, they elicit a long-lasting CCR5 internalization, resulting in a deep block of HIV infection in either CD4^+^ T lymphocytes or T cell lines [31–34]. The natural occurrence of mucosal immunoglobulin (Ig) A and systemic IgG to CCR5 in some HESN [35, 36] suggests that, similarly to the CCR5Δ32 mutation, these Abs may play a role in the protection against HIV infection/transmission (Fig. 1b). CCR5 down-regulating Igs are also found in a subset of long-term non-progressors (LTNP) [37], suggesting a role for such Abs in controlling viral replication in vivo. The mechanism induced by exposure to natural CCR5 Abs could be useful to design molecules eliciting more stable receptor degradation, thus resulting in a deep block of HIV infection for a long time.

Of note, natural IgA to CCR5, but not commercial Abs to CCR5 such as 2D7 (an HIV blocking monoclonal Ab recognizing the second extracellular loop of CCR5), specifically block HIV transcytosis (i.e., HIV transfer across mucosal membranes) in several epithelial cell lines. Thus, the blocking mechanism of anti-CCR5 Abs at mucosal membranes may differ from that exerted on CD4^+^ T lymphocytes, since HIV would bind CCR5 intracellularly in the endosomes during transcytosis. We hypothesize that natural anti-CCR5 Abs present at mucosal sites bind CCR5 and are then internalized with it, thus preventing interaction with HIV and subsequent transcytosis [38].

### The CCR2/CCL2 chemokine axis as a key driver of HIV-associated chronic inflammation

CCR2-mediated monocyte recruitment is crucial for the resistance to several viral infections [39], but it is deleterious and enhances pathology during influenza virus and HIV infections. Although CCR2 was reported to act as a co-receptor by rare HIV strains [40, 41], it is not used for cell entry in vivo and its function in the pathogenesis of HIV infection is mostly linked to the role played in leukocyte movement and inflammation, leading to the recruitment of new targets for infection in a favorable environment for viral replication.

CCR2 and CCL2 polymorphisms have been shown to affect susceptibility to HIV infection, disease progression and HIV-associated morbidities, although many works were conflicting and the mechanisms were not clear [5]. The most studied were the single nucleotide polymorphisms CCR2-V64I and CCL2-2518 A/G (alternatively designed -2578). The former was associated with a slower disease progression in some studies [42, 43]. This may be linked to the ability to heterodimerize with CCR5 and/or CXCR4 and reduce their expression. Indeed, CXCR4 can dimerize with the CCR2-V64I mutant, but not with wild-type CCR2 [44]. However, this polymorphism was not associated with altered CCR5 expression or co-receptor function in HIV-infected individuals [45]. Since the CCR2-V64I mutation tracks, through linkage disequilibrium, with mutations in the promoter region of CCR5, population-specific patterns of CCR2 and CCR5 haplotypes may also explain disparities in infection or disease progression [43, 46]. Homozygosity for the CCL2-2518 G allele, which leads to increased CCL2 expression, was associated with a 50% reduction of the risk of acquiring HIV, although after infection this genotype enhanced disease progression and the risk of HIV-associated neurocognitive disorders (HAND) [47]. CCL2 may thus partially protect from infection, but it may accelerate disease progression and increase the risk of HAND once infection is established, through its pro-inflammatory properties and ability to stimulate HIV replication. Interestingly, individuals with the -2578G allele showed higher cerebrospinal fluid (CSF) CCL2 levels, increased CSF pro-inflammatory markers and worse neurocognitive functions [48]. This allele also conferred an increased risk for atherosclerosis to HIV-infected subjects [49].

Increased CCL2 levels in the blood and CSF of HIV-infected individuals were found to correlate with viral load [50–52]. Either HIV infection or exposure to viral proteins (i.e., gp120, Nef, Tat, p17) induced CCL2 and/or CCR2 expression in different cell types [i.e., monocytes/macrophages, peripheral blood mononuclear cells (PBMCs), hepatic stellate cells (HSCs), astrocytes, microglia, endothelial cells] [5]. Interestingly, activation of CCR5 signaling by gp120 mediated CCL2 up-regulation in both macrophages and HSCs [53–55]. High CCL2/CCR2 levels in HIV^+^ subjects are tightly linked to increased inflammation/immune activation and development of co-morbidities through leukocyte recruitment and maintenance of the inflammatory status that represents a hallmark of HIV infection also in the post-HAART era [5]. These mechanisms have been extensively studied in the central nervous system (CNS) (Fig. 1a). The transmigration of HIV-infected monocytes into the CNS, mainly mediated by CCL2, transports the virus into the CNS, resulting in infection of macrophages and microglia thus contributing to the establishment and maintenance of the CNS viral reservoir and to HAND [56, 57]. The CNS infected cells release soluble factors and viral proteins, leading to additional monocytes recruitment, neuroinflammation, and neuronal damage. CCL2 levels are highly increased in brain tissues and CSF of people with HAND [52, 58] and remain elevated even with successful antiretroviral therapy [59], resulting in ongoing monocyte transmigration into the brain and chronic, low-level neuroinflammation. A mature CD14^+^CD16^+^ monocyte subset expressing high CCR2 levels was detected in individuals with HAND and proposed as a key player of HIV entry into the CNS and a peripheral blood biomarker of HAND [60, 61]. Furthermore, a higher frequency of CCR2^+^CCR5^+^ monocytes was found in symptomatic cognitively impaired HIV^+^ subjects [62].

The CCL2/CCR2 axis is also implicated in the inflammatory processes that facilitate HIV replication in mucosal tissues. The immune system of the FRT is regulated by the variations during the menstrual cycle of the sex hormones estradiol and progesterone [63]. Studies with nonhuman primates and human explant cultures have suggested the existence of a window of susceptibility for HIV infection during the luteal phase of the menstrual cycle, characterized by high progesterone levels. Very recently, an elevated cervicovaginal lavage concentration of CCL2 was found during the follicular phase of the hormonal/menstrual cycle, associated with an increase in the proportion of CCR5^+^CD69^+^CD4^+^ T lymphocyte [64]. This suggests that these highly susceptible cells may be infected during the follicular phase and their recirculation during the luteal phase may disseminate the virus, thus explaining the association of productive infection with this latter phase. Low estradiol levels in post-menopausal women may be linked to an inflammatory status that increases HIV transmission and replication. Indeed, higher levels of CCL2, associated with enhanced HIV p24 Gag release and viral transcription were found in ex vivo explants of ectocervical tissue from post- compared to pre-menopausal women, and blocking of this chemochine was shown to decrease HIV transcription [65]. Cervicovaginal lavage of women with detectable genital tract viral load contained higher CCL2 concentrations compared to those with undetectable viral load [66], further suggesting that CCL2 is a key player of HIV infection in the FRT.

CCL2 also has direct, cell type-dependent effects on viral replication (Fig. 1a). In resting CD4^+^ T cells, CCL2 exposure led to CXCR4 up-regulation, making these cells more permissive to X4 HIV infection and increasing their migration in response to gp120 [67]. This phenomenon might be particularly relevant in late disease stages when both high CCL2 levels and X4 viruses are present. In HIV^+^ CD8^+^ T cell-depleted PBMCs treated with mitogen plus IL-2 or co-cultivated with allogeneic T cell blasts of uninfected subjects, CCL2 addition stimulated HIV production in most patient’s cultures and co-cultures secreting low CCL2 levels (< 20 ng/mL). A positive correlation between the enhancement of HIV replication and CCL2 levels was observed in co-cultures. Depletion of CD14^+^ monocyte from allogeneic T blasts and addition of CCL2, respectively, down- and up-regulated virus replication during co-cultivation with CD8-depleted PBMC of HIV^+^ subjects [68]. Overall, these findings suggest that CCL2 may represent a key factor enhancing HIV spreading, particularly in anatomical sites where infection of macrophages plays a prevalent role. In these latter cells, CCL2 is produced at high levels either constitutively or following HIV infection. Blocking CCL2 activity by neutralizing Abs determined a potent restriction of HIV replication, associated with up-modulation of innate immune genes involved in the defense response to viruses, such as APOBEC3A [69, 70].

Interestingly, double CCR2^+^/CCR5^+^ immune cell populations may play key roles in HIV pathogenesis. In particular, CCR7^+^CD62L^+^ T CM lymphocytes, which represent major HIV reservoirs, were found to co-express CCR5 and CCR2 [71]. These lymphoid cells migrate in response to CCL2 and are susceptible to HIV infection. Thus, CCL2 produced early in HIV infection may recruit CCR2^+^ CM T cells to the site of inflammation, where these cells can become productively infected and produce virus, or become latently infected and contribute to the stable reservoir. A reduced susceptibility to HIV infection due to low transcriptional levels of both CCR5 and CCR2 was very recently found in a small population of HIV-infected subjects who maintained a very low level of viremia in the absence of combined antiretroviral therapy (cART) [72]. These findings support the contribution of these two chemokine receptors to seeding of the latent reservoir and suggest that antagonizing CCR2/CCL2 and CCR5 may represent an innovative effective strategy to fight latency [73].

### Targeting CCR5 and CCR2/CCL2 in HIV infection

Since CCR5-defective individuals have normal inflammatory and immune reactions, CCR5 was interpreted as a redundant and therefore dispensable molecule in adults, thus becoming a potential preventive and therapeutic target for blocking HIV entry and for immune modulation. Though several strategies to prevent CCR5 function in HIV entry were developed and tested (Fig. 1b), the small molecule antagonist maraviroc (MRV) is right now the only CCR5 targeting drug approved for clinical use [13]. Yet, as MRV did not showed better antiviral activity compared to conventional drugs, it is employed only in treatment-multiexperienced patients [74]. Moreover, HIV escape mutants to this drug were described and it was demonstrated that HIV can infect target cells by utilizing drug-bound CCR5 as well [75, 76]. A recent study demonstrated that MRV induces latent HIV transcription in resting CD4^+^ T cells from subjects on cART by activating NF-kB, thus acting as a weak receptor agonist [77]. Thus, CCR5 targeting may also represent a new latency-reversing approach to interfere with HIV persistence during antiretroviral therapy.

In recent years, monoclonal Abs (mAbs) were developed as a distinct class of CCR5 inhibitors (Fig. 1b). They potently inhibit R5 HIV in vitro, and have strong antiviral activity in HIV^+^ subjects. CCR5 mAbs offer several potential advantages over existing drugs in terms of less frequent dosing, tolerability and limited interactions with other drugs or food [78]. Two human mAbs to CCR5 (Mab004 and PRO140) are currently in clinical development in HIV infection [79]. MAb004 appeared safe and significantly reduced viral load in HIV-infected patients. PRO140, which blocks HIV entry and replication at concentrations that do not affect receptor activity, is currently in phase III clinical trial. It determined a potent short-term dose-dependent HIV RNA suppression without significant adverse events in patients. Both mAbs bind CCR5 in the HIV-binding site, thus acting with a competitive rather than allosteric mechanism, as MRV, and they did not affect lymphocyte activity [80, 81]. The use of Abs might solve the limitations of currently available therapies for HIV-infected patients, such as the complication associated with multidrug resistant viruses, drug–drug interactions and also the potential interactions with redundant chemokine receptors. Another mAb, ST6, is a CCR5 intrabody (it binds CCR5 intracellularly), which recognizes the N-terminus of CCR5 and efficiently down-regulates the receptor from the cell membrane, thus preventing HIV entry and replication [82].

Mixtures of HIV-entry inhibitors or “super Abs” binding different epitopes are emerging as more potent strategies with respect to a single inhibitor or an Ab recognizing only one epitope [80]. Furthermore, viral strains resistant to CCR5 inhibitors were more susceptible to be neutralized by cross-neutralizing mAbs compared with the parental virus, probably because drug-bound CCR5 induces virus adaptation to the new receptor conformation, thus rendering the envelope more susceptible to the neutralizing mAbs [75].

Modified chemokines, such as CCL5/RANTES analogs, represent another class of CCR5 inhibitors. In particular, PSC-RANTES, an N-terminally modified analog of RANTES, showed strong HIV inhibition, although a high concentration was needed to block viral replication in animal models and the drug-induced signaling that could increase inflammation [83]. 6P4-, 5P12-, 5P14-RANTES are modified PSC-RANTES molecules. 6P4-RANTES makes the intracellular sequestration of CCR5 longer, whereas 5P12- and 5P14-RANTES induce CCR5 internalization without signaling activity [84]. AOP-RANTES internalizes CCR5, blocks HIV infection in macrophages and inhibits CCR5 re-expression on cell membrane [85, 86].

RNA-based methods and gene editing to induce a CCR5^−^ phenotype are other approaches under clinical evaluation [87]. The more promising RNA-based strategies are RNA silencing and antisense RNAs delivered by pseudotyped lentiviral or adenoviral vectors. Three main technologies have been used to edit CCR5. Zinc finger and transcription activator-like nucleases recognize their target DNA sequence and induce a double-stranded break through Fok1 endonucleases. The clustered regularly interspaced short palindromic repeats/Cas9 system works through a guide RNA sequence that forms a complex with the Cas9 endonuclease [88]. Some of these approaches have been or are being tested in clinical trials in HIV^+^ individuals [89].

Targeting CCR2 and CCL2 is a very active area of drug development with potential application in several important acute and chronic human diseases, and it has been the subject of several reviews in recent years [5, 8, 90]. Two main strategies have been used to block CCR2/CCL2 function, namely small molecule CCR2 antagonists and CCR2 or CCL2 specific neutralizing Abs.

Cross-reactivity with the highly homologous CCR5 was the major challenge in the development of small molecule CCR2 antagonists. Indeed, one of the early CCR5 antagonists developed, TAK779, was found to be a potent antagonist of both CCR5 and CCR2 [91] and cross-reactivity with CCR5 is a common property to many CCR2 antagonists. Since this feature may be therapeutically advantageous in the treatment of complex diseases such as HIV infection and a number of inflammatory conditions, some companies have developed compounds that inhibit both receptors, such as cenicriviroc (CVC) [92].

CVC is a first-in-class dual CCR2/CCR5 antagonist that was initially developed for the treatment of HIV infection. By blocking CCR5, it inhibits HIV entry into host cells, but has also potential anti-inflammatory effect due to CCR2 inhibition. Thus, unlike MRV, in addition to the direct anti-HIV effects, CVC could be effective in treating the chronic immune activation associated with suppressed HIV infection, thus improving the management of HIV-infected patients. CVC completed phase II clinical development in HIV-infected subjects, showing favorable safety and efficacy [93]. Notably, the analysis of immune and inflammatory biomarkers revealed a decrease in sCD14 and sCD163 levels, as well as up-regulation of the defense response gene APOBEC3A, in CVC-treated individuals [93–95]. Although data from clinical trials supported its further evaluation as a backbone for new multi-drug combination therapy for HIV in phase III registration studies, CVC is not longer being developed in HIV infection.

## CCR5 and CCR2/CCL2 in the pathogenesis of MS

### Complex roles of CCR5 and CCR2/CCL2 in inflammation, axonal damage and repair in MS

MS is a heterogeneous, multifactorial disease of the CNS, and broadly comprises two main clinical stages: a relapsing–remitting (RRMS) stage, characterized by discrete episodes of neurologic dysfunction followed by clinical remission, and a progressive stage with steadily worsening disability. Progressive MS usually evolves from RRMS, although some patients may have progressive disease from onset [96, 97].

The pathologic hallmark of MS is the inflammatory lesion, characterized by leukocyte infiltration, demyelination, oligodendrocyte loss, axonal damage, and ultimately leading to the neurologic dysfunction associated with clinical relapses. To limit inflammation and initiate repair, immune-modulatory networks are triggered, which result in at least partial remyelination associated with clinical remission. The “classical active lesion” with profound inflammatory leukocyte infiltration (CD4^+^ and CD8^+^ T lymphocytes, B and B cell-derived plasma cells, macrophages) predominates in RRMS. In progressive disease, lesions tend to have an inactive core surrounded by a narrow rim of activated microglia, astrocytes and macrophages [96].

**Fig. 2:**
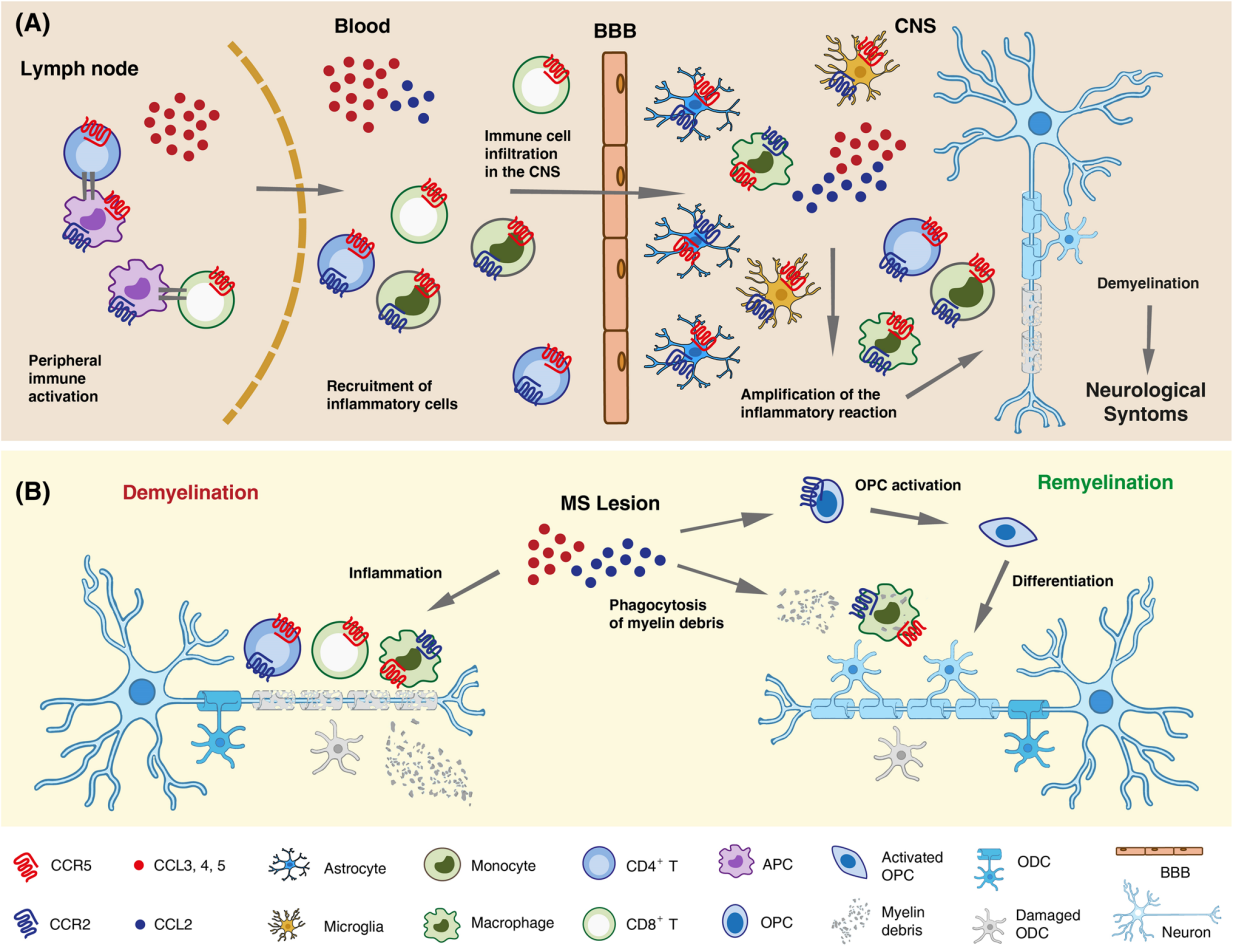
Schematic representation of the multiple roles of CCR5 and CCR2/CCL2 in inflammation, axonal damage and repair in MS. **a** In the lymph node, CCR5 ligands secreted by activated antigen-presenting cell (APCs) and CD4^+^ T helper cells mediate CD8^+^ T cells recruitment and T cell activation. CCR5 ligands also promote trans-endothelial migration and crossing of the blood–brain barrier (BBB) by circulating CCR5^+^ effector T cells and phagocytes. CNS invading Th1 cells and monocytes, as well as CNS resident innate immune cells such as microglia and astrocytes, co-express CCR2 and CCR5. Their ligands CCL2, CCL3, CCL4 and CCL5 are also up-regulated in MS lesions where they can be produced by activated microglia, astrocytes and neurons. CCL2 and CCR5 ligands secreted in situ amplify local autoimmune/inflammatory responses ultimately leading to demyelination and axonal damage. **b** Besides mediating demyelination and neuronal damage (left), CCL2 and CCR5 ligands contribute to remyelination and damage repair (right). CCR5^+^CCR2^+^ macrophages are essential for the clearance of myelin debris. CCL2 also enhances oligodendrocyte precursor (OPC) mobility, enabling them to populate demyelinated lesions, where they differentiate into myelin sheath-forming oligodendrocytes (ODC)

Of note, CNS invading Th1 cells and monocytes, as well as CNS resident innate immune cells such as microglia and astrocytes, co-express CCR2 and CCR5 [98] and several lines of evidence point to a complex role of these receptors in MS pathophysiology (Fig. 2). The disease course has been reported to be less severe in carriers of the CCR5Δ32 allele, although contrasting results have been reported on the association between CCR5Δ32 and MS risk [99–101]. Both CCR2 and CCR5 are highly expressed within and around active MS lesions, mainly in infiltrating T cells and macrophages, and in resident microglia [99, 102]. Their ligands CCL2, CCL3, CCL4 and CCL5 are also up-regulated. CCL2 immunoreactivity was mainly associated with astrocytes and macrophages within the lesion, and with hypertrophic astrocytes in the surrounding parenchyma. CCL2 expression was found to correlate with lesion activity and appears mainly restricted to white matter lesions, which contain a more massive leukocyte infiltrate than gray matter lesions [103]. In addition to leukocyte recruitment into the CNS in RRMS, CCL2 has been proposed to promote activation of microglia/macrophages and expansion of demyelinating lesions in secondary progressive MS [104].

CCR5 ligands are also present within active demyelinating plaques, although with a different spatial distribution compared to CCL2, being mainly expressed in the blood vessel endothelium, perivascular cells and surrounding astrocytes [105, 106]. CCR5 is expressed on most CD8^+^ T cells, monocytes and macrophages within inflammatory MS lesions; it could, therefore, contribute to recruitment of these cells to the inflamed tissue, their activation and/or their survival [99]. Importantly, both chemokine/receptor axes may also contribute to remyelination (Fig. 2b). Remyelinating lesions display significantly more abundant CCR5^+^ cells than demyelinating lesions, suggesting a role of these cells in the repair process [107]. Macrophages recruited via CCL2/CCR2 play essential roles in the phagocytosis of myelin debris and promote the regenerative response [108]. Furthermore, CCL2 up-regulation was described in activated oligodendrocyte progenitor cells nearby demyelinated areas, and CCL2 proved to enhance in vitro chemotaxis of these cells. It has thus been suggested that CCL2, by enhancing oligodendrocyte progenitor mobility, enable them to populate demyelinated lesions. Once within the lesion, progenitors may differentiate into mature myelin sheath-forming oligodendrocytes, thus allowing remyelination [109].

Along with their role in inflammation, both CCL2 and CCL5 have been shown, at least in mice, to regulate neuronal excitability and synaptic transmission [110–112]. Hence, they likely play an important role in the neuroimmune crosstalk, which contributes to neuronal damage associated with acute inflammation, but it is crucial for the development of compensatory neuronal plasticity as well [110].

While generally co-expressed in MS lesions, CCR2 and CCR5 ligands in body fluids of MS patients seem to follow an opposite trend of regulation. Almost unique among inflammatory chemokines, generally up-regulated, CCL2 is consistently present at lower levels in the CSF (and often in the blood) of MS patients compared to healthy controls (HC) or patients with other non-inflammatory diseases. The more active/inflammatory the disease stage is, the lower the CCL2 concentration will be (i.e., CCL2 levels in relapse < remission < progressive MS < HC or non-inflammatory diseases) [113]. CCL2 concentration in the CSF is higher than in serum, indicating an intrathecal production, and CSF CCL2 inversely correlated with other markers of CNS inflammation as well as with the level of the neurofilament light protein, a marker for axonal damage [114, 115]. Conversely, CCL5 concentration in the blood and in the CNS of MS patients directly correlates with disease activity and inflammation, with the highest levels of CCL5 found during relapses. A corresponding enrichment in the intrathecal compartment (CSF and brain lesions), of CCR5^+^ cells, particularly CD8^+^ T lymphocytes, DCs and monocytes was observed [99, 116]. Interestingly, a unique population of CCR2^+^CCR5^+^ T cells was found selectively enriched in the CSF of MS patients during relapse but not in patients with other neurologic diseases and proposed as a therapeutic target. This Th1 subset produced high levels of two proteins involved in the CNS pathology, matrix metalloproteinase-9 and osteopontin, which showed high invasive potential across an in vitro blood–brain barrier model and was reactive to myelin basic protein, one of the putative MS autoantigens [117]. Disease-modifying therapies for MS affect the endogenous availability of CCR2 and CCR5 ligands in CNS. Following treatment with methylprednisolone, widely used to accelerate relapse recovery, blood CCL2 increased and CCL5 decreased in parallel with normalization of inflammation markers [102]. A decrease of CCR5 expression in CD4^+^ cells in the CSF was also reported [118]. CCL2 is strongly inducible by IFN and serum CCL2 levels are consistently higher in MS patients undergoing IFN therapy compared to untreated patients [119–122]. It was hypothesized that IFN-induced chemokine up-regulation in the periphery could desensitize chemokine receptors on leukocytes or cause a “reverse-gradient” which neutralize leukocyte recruitment into the CNS. However, CCL2 plasma levels during long-term IFN treatment of MS patients did not predict therapeutic response at 1 or 2 years of therapy, thus questioning the relevance of CCL2 induction as a main mediator of the therapeutic action of IFN [122].

In conclusion, although a pathogenic role of CCL2/CCR2 as well as of CCR5 and its ligands in MS is widely recognized, mainly due to their role in leukocyte recruitment to active lesions and amplification of the local inflammatory response, the role of these chemokine/receptors axes in MS is likely much more complicated.

### Targeting CCR5 and CCR2/CCL2 in MS

In recent decades, better understanding of mechanisms underlying RRMS has led to the development of immunosuppressive and immune-modulating therapies that reduce both severity and frequency of new relapses. Many of the current therapies actually target inflammation and leukocyte trafficking in the CNS. In this context, some CCR2 targeting drugs have entered in clinical trials for MS in past years. They generally showed a favorable safety profile, but failed from a therapeutic point of view and are not currently involved in clinical studies [5, 123]. A potent CCR2 antagonist, MK-018, showed good pharmacokinetic profiles in preclinical studies and demonstrated efficacy in animal models. This drug entered in phase II clinical trials for MS, but no significant improvement compared with placebo was reported [124].

The humanized anti-CCR2 mAb MLN1202 was also tested in MS patients and reported to be effective in reducing the number of lesions in the brain [125]. However, the compound was no longer developed for such indication, suggesting that the activity may be insufficient to compete with current therapies.

The use of MRV was proposed to manage the progressive multifocal leukoencephalopathy (PML) and subsequent immune reconstitution inflammatory syndrome (IRIS) that are among the most concerning side effects associated with MS immunotherapies, in particular with natalizumab. The rationale is the presence of CD8^+^ T cells expressing high CCR5 levels in the CNS inflammatory infiltrate that results from PML Furthermore, MRV is assumed to readily penetrate the blood–CSF barrier [99]. Although initial case reports suggested that MRV could be beneficial in PML–IRIS management, a subsequent report of three cases with no clear clinical effect of the drug questioned its use in MS [99, 126, 127].

The past 20 years have witnessed remarkable and unprecedented advances in the treatment of RRMS, However, most of them are not effective for progressive MS, whose treatment remains a major challenge in MS research. The pleiotropic effects of chemokines, including CCL2 and CCR5 ligands, in the neuro-immune crosstalk and in neurodegeneration remains to be fully elucidated and may constitute an avenue for future research to address this challenge.

## CCR5 and CCR2/CCL2 in the pathogenesis of liver disease

### CCR2/CCL2 and CCR5 as drivers of hepatic fibrosis and HCC development

**Fig. 3:**
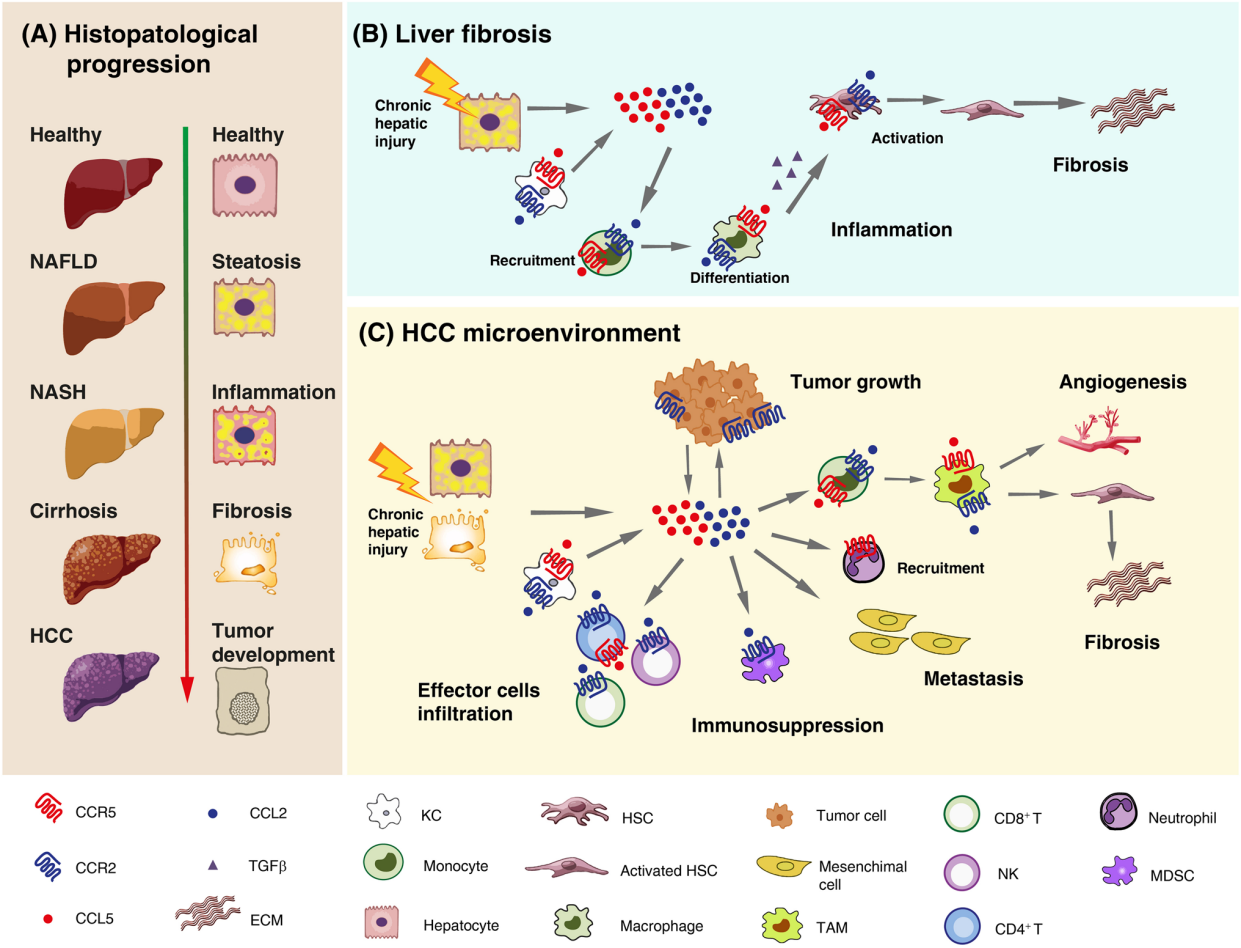
Schematic model of the role of CCR5 and CCR2/CCL2 in liver fibrosis and tumor development. **a** Hepatocyte histological changes during the progression from a healthy liver to non-alcoholic fatty liver disease (NAFLD), non-alcoholic steatohepatitis (NASH), cirrhosis and hepatocellular carcinoma (HCC). **b** Contribution of CCR5 and CCR2/CCL2 to the major pathogenic events leading to liver inflammation and fibrosis. Activated Kupffer cells (KCs) and damaged hepatocytes secrete CCR2 and CCR5 ligands, which mediate monocyte recruitment, their differentiation into macrophages, and activation of hepatic stellate cells (HSCs), thus contributing to inflammation and extracellular matrix (ECM) deposition. **c** Contribution of CCR5 and CCR2/CCL2 to HCC progression. In the tumor microenvironment, CCR2 and CCR5 ligands secreted by KCs, damaged hepatocytes and tumor cells promote tumor growth and mediates monocyte recruitment and their maturation into tumor-associated macrophages (TAMs) with pro-angiogenic and pro-fibrotic features; the infiltration of myeloid-derived suppressor cells (MDSCs), thus contributing to immunosuppression; the progression of primary tumors towards metastases by promoting the migration, invasion and epithelial–mesenchymal transition of HCC cells. On the other hand, these chemokine axes also promote the infiltration of effectors cells, which contributes to tumor eradication

Liver fibrosis is a response to hepatic insults that occurs in most types of chronic liver diseases, most importantly alcoholic and non-alcoholic fatty liver diseases (ALD and NAFLD) as well as viral hepatitis [128]. NAFLD is the most common liver disease in industrialized countries, and especially its progressive inflammatory form, non-alcoholic steatohepatitis (NASH) predisposes to cirrhosis and HCC (Fig. 3a) [129]. NAFLD and NASH are commonly associated with obesity-related disorders (e.g., type 2 diabetes mellitus and metabolic syndrome), and due to the rapidly increasing frequencies of these conditions, they are projected to become an enormous clinical and economic burden [130]. Hepatic fibrosis, characterized by excessive deposition of extracellular matrix (ECM) proteins, is the major feature predicting liver-related and overall mortality in patients with NASH. The accumulation of fibrogenic macrophages in the liver is the major central pathway driving fibrosis progression identified in patients and mouse models of liver fibrosis [131]. Hepatic macrophages are heterogeneous cell populations, consisting of liver resident phagocytes, termed Kupffer cells (KCs), and monocyte-derived macrophages, which represent the dominant macrophage population. During acute or chronic liver injury, circulating monocytes are massively attracted to sites of hepatic injury, where they differentiate into liver macrophages that promote the activation of HSCs to become myofibroblasts, the main source of ECM, especially collagen types 1 and 3, in the chronically inflamed liver. Hepatocytes, KCs and infiltrating monocytes/macrophages are major producers of TGF-β, a key fibrogenic cytokine promoting collagen production by activated HSCs [132]. In addition, HSCs secrete several cytokines and chemokines that amplify and maintain the inflammatory response.

Recruitment of extra-hepatic inflammatory cells to the site of hepatic injury is largely mediated by chemokines and their receptors [133]. Monocytes, KCs, HSCs and damaged hepatocytes express CCR2 and CCR5 on their surface. Increasing evidence implicates these receptors and their ligands CCL2 and CCL5, secreted by various liver cells like activated KCs or damaged hepatocytes, in the pathogenesis of liver fibrosis through promotion of monocyte/macrophage recruitment and tissue infiltration, as well as HSC activation following liver injury (Fig. 3b). In mouse models of hepatic fibrosis, either targeted deletion or pharmacological inhibition of CCR2 or CCR5 resulted in lower immune-cell activation and reduced liver fibrosis [134, 135]. Although many of the mechanistic concepts are derived from animal models of NASH and liver fibrosis, the same pathways were shown to be active in human disease. For instance, CCR2/CCL2 is up-regulated in fibrotic livers from patients, alongside an accumulation of inflammation-polarized monocyte-derived phagocytes that activate HSCs [136]. Patients with NAFLD have increased levels of the macrophage activation marker sCD163, underlining the importance of macrophages in chronic liver injury [137]. Furthermore, increased proportions of CCR2^+^ macrophages in visceral adipose tissue are associated with histological disease severity of NASH in obese patients [138].

Interestingly, NAFLD frequency is higher in HIV-infected patients (30–40%) than in the general population (14–31%) [139]. Although the definition of NAFLD excludes viral hepatitis, hepatic steatosis is a common condition in HCV-infected subjects and its prevalence is even higher in HIV/HCV-coinfected individuals (30–70%). In these patients, the interactions among HIV infection, viral hepatitis and steatosis accelerate the progression to hepatic fibrosis, resulting in an earlier appearance of end-stage liver disease. HIV gp120 was shown to modulate different aspects of HSC biology, including stimulation of migration and increased expression of type I procollagen and pro-inflammatory cytokines such as CCL2. These actions are mediated via activation of CCR5 and are blocked by a CCR5 antagonist [55]. These data suggest a direct role of HIV in the process of hepatic fibrogenesis.

Chronic liver disease and cirrhosis associated with viral hepatitis and excessive alcohol intake are the most important risk factors for HCC development. However, NAFLD- and NASH-related HCC greatly increased in recent years and it is anticipated to rise exponentially due to the growing epidemic of obesity and diabetes [140]. HCC is among the most lethal and prevalent cancers in the human population. Its development is closely related to the presence of chronic liver disease and is a complex multistep process that involves sustained inflammatory damage, hepatocyte necrosis and regeneration, fibrotic deposition, and genomic alterations [141].

The chemokine system plays a fundamental role in hepatocarcinogenesis by modulating the immune response in the tumor microenvironment (TME) and directly affecting HCC cell growth, invasion and migration properties [133, 142]. As discussed previously in this paragraph, CCR2, CCR5 and their ligands are key players in orchestrating the interaction among parenchymal liver cells, KCs, HSCs, and infiltrating immune cells during liver inflammation (Fig. 3b). These cellular interactions result in the remodeling of the hepatic microenvironment toward a pro-inflammatory, pro-fibrotic, pro-angiogenic and thus pre-neoplastic milieu. Once developed, liver neoplasms provoke pro- and anti-tumor immune responses that are also critically regulated through differential activation of chemokine networks.

In HCC tissues, CCR2, CCR5 and their ligands are expressed by tumor as well as non-tumor cells and are modulated by inflammatory cytokines. Enhanced CCL2 expression in human liver cancer was reported to represent an independent prognostic indicator in patients with HCC and was linked to a decreased survival rate [143]. In addition, the CCR2-64I gene polymorphism was shown to be an important factor for HCC susceptibility, but it did not influence the clinical progression of HCC [144].

An increased presence within the HCC TME of tumor-associated macrophages (TAMs), which mediate fibrogenesis and angiogenesis, has been consistently associated with poor patient prognosis. The CCL2/CCR2 axis plays a fundamental role in monocyte recruitment and their maturation into TAMs (Fig. 3c). Bartneck et al. dissected the TAM subtypes, particularly those mobilized by CCL2/CCR2, involved in fibrogenesis-driven hepatocarcinogenesis. They found a specific accumulation of CCR2^+^ TAMs at the stroma/tumor interface in resected human HCCs, where they co-localize with endothelial cells in areas of intense vascularization. These TAMs did not belong to the suppressive M2-like population, but to an M1 population showing an inflammatory and pro-angiogenic polarization. In a mouse model of liver fibrosis and hepatocarcinogenesis, CCL2 inhibition by an RNA aptamer resulted in reduced TAM1 liver infiltrate and pathogenic angiogenesis, improvement of tissue fibrosis, and a significant inhibition of tumor progression [145]. In addition to TAMs, the CCL2/CCR2 axis can drive the infiltration of myeloid-derived suppressor cells, thus contributing to increased immunosuppression in the TME [146, 147]. Finally, CCL2 was demonstrated to promote the migration, invasion and epithelial–mesenchymal transition (EMT) of HCC cells, which is implicated in the progression of primary tumors towards metastases [148, 149]. On the other hand, CCL2 expression in human HCC directly correlates with the infiltration of effectors cells, such as CD4^+^ Th1, CD8^+^ and NK cells, which contributes to tumor eradication [150]. These observations confirm that chemokines can have a dual role (suppression and activation) on immune cells in the HCC microenvironment. CCL2 also influences the growth of HCC cells. In particular, CCL2/CCR2 over-expression promoted the proliferation of HCC cells in response to apigenin or co-culture with cancer-associated fibroblasts [151, 152].

CCR5-mediated inflammation is as well important in hepatocarcinogenesis. The CCL5–CCR5 axis participates in the development of HCC, and CCL3 and CCL4 show a definitive role in accelerating the course of HCC [153]. CCL3, which is remarkably increased in different HCC cell lines when stimulated with IL-1α or IL-1β, may attract a large amount of macrophages and neutrophils into the inflammation sites. CCR5 has been demonstrated to play a critical role in both the development and progression of liver cancer. Indeed, in a mouse model of diet-induced HCC, treatment with MRV determined significantly higher survival rates, less liver fibrosis, lower levels of liver injury markers and chemokines, less apoptosis and proliferation, as well as a lower tumor burden compared to controls [154]. Likewise, in the same mouse model, CCR5 knockout was shown to significantly reduce macrophage recruitment and trafficking to the liver, inflammation, and periductal accumulation of oval cells, which are the putative liver progenitor cells that proliferate and differentiate in response to liver damage, thus resulting in abrogation of fibrosis and significant decrease in tumor incidence and size [155].

## Targeting CCR2/CCL2 and CCR5 in NASH and HCC

Despite its rising prevalence, there are currently no approved treatments for NASH. Targeting central pathways driving fibrosis progression, such as CCR2/CCR5-mediated accumulation of fibrogenic macrophages in the liver, might provide therapeutic opportunities for the therapy of NAFLD/NASH. Thus, CCR2 and CCR5 have become promising targets for anti-fibrotic therapy.

A number of studies demonstrated that CVC displayed anti-inflammatory and anti-fibrotic effects across a range of in vivo animal models, including liver fibrosis, NASH, ALD and kidney fibrosis [156, 157]. Therefore, the drug was investigated in a phase II clinical trial (CENTAUR; NCT02217475) and is currently being evaluated in a phase III study in patients with NASH and fibrosis (NCT03028740). Data from CENTAUR suggested that NASH patients who received CVC have a greater likelihood of a sustained reduction in liver fibrosis over 2 years compared with those who received placebo [158]. Other agents with different mechanisms of action are under investigation in NASH. Combination therapies targeting both inflammatory and metabolic pathways in NASH might represent valuable therapeutic options to further improve treatment outcomes, and are under evaluation in a phase II study (NCT03517540).

HCC is a chemotherapy-resistant tumor that is most frequently diagnosed at advanced stages with limited treatment options and is thus associated with a high mortality rate. Therapies for HCC are dependent on disease stage. In early stages, surgery represents the standard treatment with a 5-year survival rate in 70% of treated patients [159]. When surgery or liver transplantation is not applicable, second-line loco-regional therapies have highly variable 3–5-year survival rates [160]. In advanced unresectable HCC, the inhibitor of tyrosine protein kinases, sorafenib is the only approved systemic therapy, providing a very limited survival benefit [161]. Interestingly, CCL2/CCR2 targeting by CCR2 knockdown, CCR2 antagonists, neutralizing Abs, or RNA aptamers has been shown to inhibit malignant growth and metastasis, reduce postsurgical recurrence and enhance survival in different HCC models [143, 156, 162, 163]. In such a scenario, immunotherapeutic strategies targeting the chemokine system, alone or in combination with standard chemotherapy, may improve clinical outcome in HCC patients [164–166]. In this regard, a pre-clinical study demonstrated that a CCR2 antagonist shows anti-cancer effects increasing CD8^+^ T cells via blocking tumor-infiltrating macrophage-mediated immunosuppression, and that the anti-tumor effect was improved by combining the antagonist with low-dose sorafenib [167, 168].

## Conclusions and future perspectives

CCR5 and CCR2 are important mediators of leukocyte trafficking in inflammatory processes. The emerging evidence of their role not only in HIV infection, but also in several human inflammatory diseases and cancers, led to a growing interest in CCR5 and CCR2 therapeutic targeting. Specific small-molecule antagonists and mAbs are feasible therapeutic options to interfere with these chemokine axes, and phase I–II trials have demonstrated their safety in humans. Since in some complex disorders either CCR5 or CCR2 have emerged as key drivers of disease pathophysiology, drugs targeting both receptors represent a novel and promising way to generate effective therapeutics. The simultaneous targeting of two different disease molecules with one drug makes development less complex from a technological and regulatory perspective, because manufacturing, preclinical and clinical testing are reduced to a single, bispecific compound. Dual receptor chemical antagonists are currently the only available tools to simultaneously interfere with CCR5 and CCR2, and one of these compounds is in active clinical trials in NASH patients.

During the past decade, dual targeting with bispecific Abs has emerged as an alternative to monospecific Ab combination therapy. A substantial breakthrough was made thanks to promising clinical trial results of some bispecific Abs and development of new formats, which largely ease manufacturing and physicochemical property challenges encountered by early formats. Bispecific Abs targeting CCR5 and CCR2 may have several advantages over chemical antagonists. In particular, they can be engineered to be specific and with similar strong neutralizing activity for both receptors, and to extend their half-life even to months. This latter is an important tool to develop long-acting therapies that could be taken once a month or even less, thus improving patients’ quality of life and adherence. Abs may also elicit additional strong immune responses through Fc-mediated complement-dependent cytotoxicity or Ab-dependent cellular cytotoxicity, thus enhancing in vivo efficacy at least in some pathological conditions. Compared to the monospecific mAbs already in clinical development, bispecific mAbs would preferentially target cells co-expressing both receptors. Interestingly, double CCR5^+^CCR2^+^ immune cell populations were described in both HIV and MS patients, and they were proposed as relevant drivers of disease pathogenesis. In HIV infection, the simultaneous inhibition of CCR5 and CCR2 may offer a new chance to tackle viral persistence and inflammation and could be exploited as the component of complex therapeutic approaches aimed at a functional cure. Conversely, although a pathogenic role of CCR2 and CCR5 networks in MS is widely recognized, a deeper mechanistic understanding of their functions is still needed before implementing novel anti-chemokine strategies in patients. Targeting CCR5/CCR2 pathways might be also promising to complement conventional surgery-based and chemotherapeutic approaches in HCC. Since these chemokine networks mediate hepatic inflammation, fibrosis, angiogenesis, and EMT of hepatic tumor cells, their targeting might be useful in the prevention of cancer in patients with chronic liver diseases. In the forthcoming years, results from the undergoing phase III clinical trial with CVC in NASH patients will hopefully confirm the therapeutic efficacy observed in phase II studies on a large number of patients and will also demonstrate the true impact of CCR5/CCR2 inhibition on HCC development.
